# Comparative Study between the Diagnostic Effectiveness of Brain SPECT with [^123^I]Ioflupane and [^123^I]MIBG Scintigraphy in Multiple System Atrophy

**DOI:** 10.3390/biomedicines12010102

**Published:** 2024-01-03

**Authors:** Javier Villena-Salinas, Simeón José Ortega-Lozano, Tomader Amrani-Raissouni, Eduardo Agüera-Morales, Javier Caballero-Villarraso

**Affiliations:** 1 Nuclear Medicine Service, Virgen de la Victoria University Hospital, 29010 Málaga, Spain; villenajavier@outlook.es (J.V.-S.); simeon.ortega.sspa@juntadeandalucia.es (S.J.O.-L.); tomader.amrani.sspa@juntadeandalucia.es (T.A.-R.); 2 Neurology Service, Reina Sofia University Hospital, 14004 Cordoba, Spain; doctoredu@gmail.com; 3 Maimónides Biomedical Research Institute of Córdoba (IMIBIC), 14004 Córdoba, Spain; 4 Clinical Analyses Service, Reina Sofía University Hospital, 14004 Córdoba, Spain; 5 Department of Biochemistry and Molecular Biology, Universidad of Córdoba, 14071 Córdoba, Spain

**Keywords:** multiple system atrophy, dysautonomia, diagnostic accuracy, Ioflupane-123, meta-iodobenzylguanidine-123

## Abstract

Background: Multiple system atrophy (MSA) is a neurodegenerative disease. It has a fast progression, so early diagnosis is decisive. Two functional imaging tests can be involved in its diagnosis: [^123^I]Ioflupane SPECT and [^123^I]MIBG scintigraphy. Our aim is to comparatively analyze the diagnostic performance of both techniques. Methods: 46 patients (24 males and 22 females) with MSA underwent [^123^I]Ioflupane SPECT and [^123^I]MIBG scintigraphy. In each of these techniques, qualitative assessment was compared with quantitative assessment. Results: SPECT visual assessment was positive in 93.5% of subjects (S = 95.24%; PPV = 93.02%). A cut-off of 1.363 was established for overall S/O index (S = 85.7%, E = 100%). Visual assessment of scintigraphy was positive in 73.1% (S = 78.57%, PPV = 94.29%). For the delayed heart/medistinum ratio (HMR) a cut-off of 1.43 (S = 85.3, E = 100%) was obtained. For each unit increase in delayed HMR, the suspicion of MSA increased by 1.58 (OR = 1.58, *p* < 0.05). The quantitative assessment showed an association with the visual assessment for each technique (*p* < 0.05). Conclusions: Both tests are useful in MSA diagnosis. Comparatively, we did not observe a clear superiority of either. Striatal and myocardial deterioration do not evolve in parallel. Qualitative assessment is crucial in both techniques, together with the support of quantitative analysis. Delayed HMR shows a direct relationship with the risk of MSA.

## 1. Introduction

Multiple system atrophy (MSA) is a neurodegenerative disease belonging to the group of α-synucleinopathies. It is due to cytotoxicity induced by the accumulation of misfolded α-synuclein protein in the cytoplasm of oligodendrocytes and neurons of the central nervous system (CNS) and autonomic nervous system (ANS) [1,2]. 

At the CNS level, the substantia nigra, basal ganglia, inferior olivary nucleus, brainstem pons, and cerebellar Purkinje cells (striatonigral and olivopontocerebellar systems, respectively) are affected. The ANS is also involved at both the supraspinal (dorsal motor nucleus of the vagus nerve, locus coeruleus, and ventrolateral catecholaminergic neurons of the bulb) and medullary (intermediolateral columns and Onuf’s nucleus) levels [3,4]. All this gives rise to a wide variety of symptoms and signs.

It has been considered that in MSA, two degenerative processes occurring in parallel coexist: (i) the accumulation of α-synucleins in the nucleus and cytosol of neurons, leading to primary neurodegeneration; (ii) in turn, cytoplasmic inclusions in glial cells, leading to secondary neuronal degeneration [5]. Such changes at the cellular and tissue level would occur years before the onset of symptoms [6]. Furthermore, since there is no disease-modifying treatment available (much less curative), the disease progresses rapidly towards death, with a very poor survival rate [7,8].

The estimated global incidence of MSA is 0.6–0.7 cases per 100,000 individuals per year, with ranges varying from a minimum to a maximum of 0.1–3 cases per 100,000 people per year, respectively [9,10]. Data collected to date indicate that the incidence increases with the patients’ age, with rates reaching 3 cases/100,000 population per year in those over 50 years and 12 cases/100,000 population per year in those over 70 years [10]. The prevalence ranges from 2 to 5 cases per 100,000 population and can reach 7.8 cases per 100,000 population in individuals over 40 years [10,11]. However, it is estimated that up to 10% of patients with parkinsonism symptoms may have an underlying MSA, potentially raising these numbers to 16.4 cases per 100,000 population [4]. Currently, there are no known risks or protective factors. The scientific literature reports dissimilar results without a clear relationship to reported environmental agents [12]. While it is primarily a sporadic disease, there are reported cases with a probable familial association of autosomal recessive inheritance [10,13,14,15]. The average age of onset ranges from 54 to 60 years old [16,17,18,19,20]. This condition affects males and females similarly, with no ethnic superiority. It is diagnosed 2 to 9 times more often in men than in women, believed to be due to their higher likelihood of seeking medical attention for erectile dysfunction, which is an early symptom of this disease [21,22].

MSA can be subdivided based on the predominant symptoms at the time of the initial diagnostic evaluation, into MSA-P if parkinsonian symptoms predominate, or MSA-C when cerebellar involvement is more pronounced. Currently, this entity is understood dynamically, meaning that this subdivision may change based on the patient’s clinical evolution [23]. There is a predominance of MSA-P in Europe and the USA and MSA-C in Asia. In Spain, contrary to the rest of the studied European countries, there appears to be a majority of MSA-C [7,10,19,24]

The predominant tremor in MSA is action and postural, spasmodic, and irregular, with resting tremor observed less frequently [17]. The slowness in movements, to a greater or lesser extent (akinesia or bradykinesia), along with rigidity, worsens rapidly and progressively as the disease advances. Characteristics include falls due to postural instability, often preceding motor symptoms. Mid-term consequences include postural abnormalities such as severe trunk flexion (camptocormia) and anteriorization of the head (antecollis) [25,26,27]. Less frequently, orofacial dystonias or dyskinesias may occur, occasionally resembling sardonic laughter (a distorted facial expression due to involuntary spasms, typical in patients with *Clostridium tetani* infection) or Pisa syndrome, characterized by subacute axial dystonia causing severe lateral flexion of the head and neck, as well as axial trunk rotation. Moreover, the extension of degeneration at the putaminal level is directly associated with the lack of an adequate response to levodopa treatment [28]. The involvement of the olivopontocerebellar system is marked by gait and limb ataxia, ataxic dysarthria, dysdiadochokinesia, and scanning speech. Additionally, ocular abnormalities such as nystagmus with gaze-evoked and dysmetria may develop [25]. The neurodegeneration of the corticospinal system is also common in this disease, although its involvement is not considered a determining factor in current diagnostic criteria. Among the pyramidal signs specific to MSA patients is the extensor plantar response [23,29]. The mechanisms most frequently implicated in the development of autonomic symptoms (dysautonomia) are directly related to the pontine micturition center and the Onuf nucleus at the sacral spinal cord level. On the one hand, there is a loss of inhibitory signal input at the pontine micturition center, leading to detrusor muscle hyperreflexia. On the other hand, there is a loss of corticotropin-releasing factor neurons in the pontine micturition area and bladder. Consequently, urinary abnormalities such as incontinence, nocturia, urgency, and polyuria are typical. Severe damage to the Onuf nucleus in the sacral spinal cord causes bladder atony and erectile dysfunction in men [30,31]. The loss of catecholaminergic neurons in the C1 area of the ventrolateral medulla manifests as severe variability in blood pressure and heart rate, potentially leading to orthostatic hypotension, syncope, or postprandial hypotension [30]. The loss of mesopontine cholinergic neurons, along with degeneration at the locus ceruleus (with preservation of those in the rostral raphe), may contribute to the development of REM Sleep Behavior Disorder, typically associated with prodromal phases of other α-synucleinopathies [32,33]. Other manifestations secondary to brainstem involvement include respiratory abnormalities such as laryngeal stridor and dysfunction in temperature regulation [25,30,34].

Because of these peculiarities, this entity can be difficult to diagnose. The diagnosis is made in relation to a series of items, known as Gilman’s criteria [23,29], which were revised recently [35]. It is interesting to know which diagnostic tests would allow the identification and diagnosis of patients with MSA in the absence of post-mortem confirmatory histopathological study. In this context, currently available functional neuroimaging tests can help. In our work, we address two nuclear neuroimaging tests: SPECT of presynaptic dopamine transporters with [^123^I]Ioflupane (previously included in the 2008 criteria for possible MSA-C, but not in the current criteria) and cardiac innervation scintigraphy with [^123^I]MIBG (which is still included as diagnostic support). 

**Figure 1:**
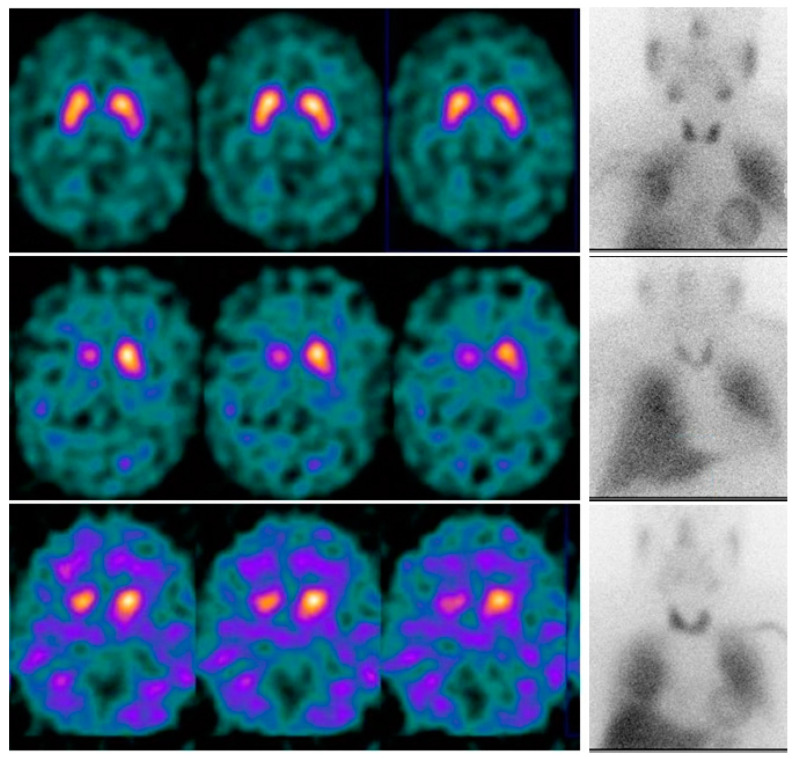
Combination of [^123^I]Ioflupane SPECT and [^123^I]MIBG scintigraphy in the study of parkinsonism. Top row: healthy patient: preserved uptake in both striatal nuclei and integrity of myocardial sympathetic function. Middle row: patient with PD: bilateral asymmetric striatal hypocaptation and absence of myocardial sympathetic function. Bottom row: patient with MSA-P: bilateral striatal hypocaptation (more symmetrical) with marked presence of cortical nonspecific activity and intact or slightly decreased myocardial sympathetic function.

The [^123^I]Ioflupane SPECT test reflects the density of presynaptic dopamine transporters in the nigrostriatal pathway (substantia nigra and caudate and putamen nuclei). It consists of a qualitative assessment of the image (visually performed by the nuclear medicine physician) which can be assisted by the quantitative assessment performed by specific software. The result will typically show bilateral striatal hypocaptation, usually more symmetrical with respect to other entities such as Parkinson’s disease (PD), and the presence of non-specific cortical activity (background) may also be observed [36]. In contrast, cardiac innervation scintigraphy with [^123^I]MIBG reflects the state of the ANS by binding to the noradrenaline transporters expressed in the presynaptic terminals of postganglionic sympathetic neurons. Similar to SPECT, the result consists of a visual and a quantitative assessment, by means of the early heart/mediastinum ratio (HMR), delayed HMR, and washout percentage [37]. In MSA, scintigraphy usually shows normal myocardial uptake of the radiopharmaceutical or slightly decreased uptake [38]. This reduction is usually of a lesser extent since the development of dysautonomia in MSA is primarily due to presynaptic dysfunction, with postsynaptic adrenergic fibers being relatively unaffected [4,39,40]. On the contrary, in other diseases (as it happens in PD) the uptake is null [10,41]. The combination of these findings is shown in Figure 1.

Moreover, while in PD the nigrostriatal involvement occurs at the presynaptic terminal, in atypical parkinsonisms (such as MSA) the involvement also occurs at the postsynaptic terminal [42]. Currently, it is not known whether pre- or postsynaptic involvement occurs earlier in MSA [43]. In view of the above, both neuroimaging studies are recommended in the most recent guidelines for the diagnosis of atypical parkinsonism [44]. The aim of this study is to analyze the diagnostic performance of both tests in MSA.

## 2. Materials and Methods

Single-center retrospective observational study of patients diagnosed with MSA—by neurologists from the movement disorders unit—who underwent SPECT of presynaptic dopamine transporters with [^123^I]Ioflupane and scintigraphy with [^123^I]MIBG, between 2004 and 2020. Both studies were conducted with a maximum one-month difference, following the request from the referring physician, typically after the initial visit to the movement disorders unit, resulting in an early diagnosis for most patients. All imaging studies were individually and blindly analyzed by three medical nuclear medicine specialists dedicated to the field of neurology. The diagnostic performance of both imaging techniques in MSA is addressed comparatively. In turn, in each of them, the effectiveness of qualitative (visual) versus quantitative analysis is confronted.

Myocardial innervation scintigraphy was obtained using a Siemens^®^ gamma camera, model Symbia^®^ (Erlangen, Germany), equipped with a dual head and low-energy, high-resolution collimator. Two static planar images were acquired in anterior chest projection with a 256 × 256 matrix at 15 min and 4 h after intravenous administration of the radiopharmaceutical [^123^I]MIBG. Subsequently, a qualitative assessment (by the nuclear physician) of myocardial uptake was performed, as well as a quantitative analysis (according to the value emitted by the device). For the latter, myocardial uptake was compared with mediastinal uptake in the 4 h image, using regions of interest (ROIs), and the delayed heart/mediastinum ratio (HMR) was obtained [44].

$$\mathrm{Heart}/\mathrm{mediastinum}\;\mathrm{ratio}\;(\mathrm{HMR})=\frac{Mean\;counts\;per\;pixel\;in\;myocardium}{Mean\;counts\;per\;pixel\;in\;mediastinum}.$$

The [^123^I]Ioflupane SPECT was used with the same gamma camera and collimator described. Tomographic images of the skull were obtained 3–4 h after administration of the radiopharmaceutical and after thyroid block with Lugol’s solution. A 360° circular orbit was performed around the skull, with 3° intervals, acquiring 60 images with a duration of 35 s per interval, with a matrix of 128 × 128. Subsequent reconstruction of the images was performed using filtered back projection algorithms without attenuation correction (Hanning filter with a frequency of 0.7). Images were analyzed according to transaxial slices and orbito-meatal orientation, consisting of a qualitative assessment of the same and a quantitative assessment using ROIs to compare the average number of striate nuclei counts with respect to the occipital lobe, known as the striate/occipital index (S/O). 

### 2.1. Inclusion Criteria

Both techniques included patients with suspected or clinical diagnosis of MSA, referred to the nuclear medicine department. 

### 2.2. Exclusion Criteria

**Figure 2:**
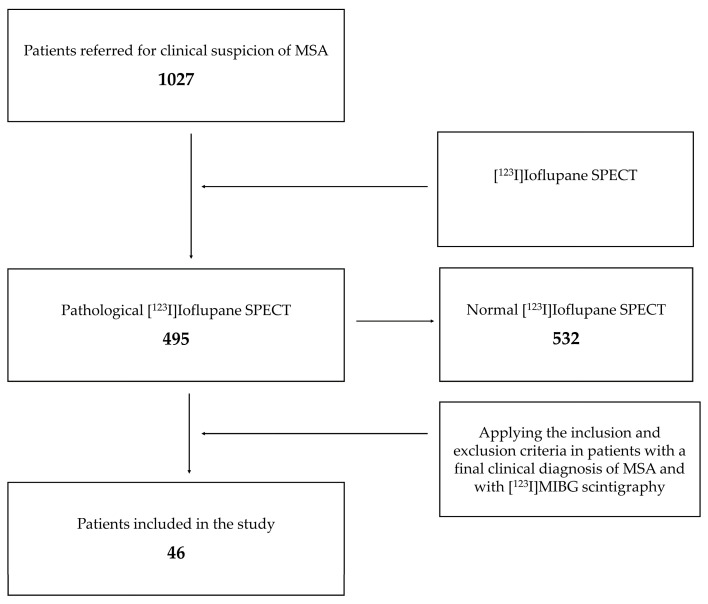
Flowchart of the patient inclusion process. SPECT: single-photon emission computed tomography; MSA: multiple system atrophy.

Patients with factors that could affect both studies (qualitatively or quantitatively). (1) For [^123^I]Ioflupane SPECT: patients with medication such as antidepressants or other substances (such as cocaine). (2) For [^123^I]MIBG scintigraphy: drugs that interfere with the noradrenaline transporter and its vesicular storage, and diseases and common causes of small fiber neuropathies (such as diabetes mellitus) (Figure 2). 

### 2.3. Ethical Considerations

Authorization was obtained from the Biomedical Research Ethics Committee of the province of Málaga. Prior to the performance of both neuroimaging tests, informed consent was obtained from each patient. The harmonized tripartite standards of the Helsinki declaration, the Organic Law on Biomedical Research of 15/2007 of 3 July, the Organic Law on Personal Data Protection (LOPD) of 13 December 2018, the code of ethics of the “Organización Médica Colegial” (OMC), the basic regulatory law 41/2002 on patient autonomy and rights and obligations regarding clinical information and documentation, of November 14, as well as the standards of good clinical practice, were respected.

### 2.4. Statistical Analysis

Within the descriptive study, qualitative variables were shown as absolute and percentage frequencies and quantitative variables as mean and standard deviation. The Shapiro–Wilk test was used to determine whether the values of each variable followed a normal distribution. To establish associations between quantitative variables, the Student *t*-test (parametric) and Mann–Whitney–Wilcoxon U test (nonparametric) were used, and for qualitative variables, the Chi-squared test was applied (with Fisher’s correction when necessary). Spearman’s correlation coefficient was used to analyze possible associations between continuous quantitative variables. ROC curves were constructed to establish an optimal cut-off point for these associations. A value of *p* < 0.05 was considered significant.

## 3. Results

A total of 46 patients were analyzed: 24 males (52.2%) and 22 females (47.8%). The mean age was 63 ± 9 years (range 47 to 79 years). They were classified according to Gilman’s criteria into 33 MSA-probable (71.7%) and 13 MSA-possible (28.3%). The predominant MSA subtype was MSA-parkinsonian (MSA-P) (80.4%) versus MSA-cerebellar (MSA-C) (19.6%). In total, 30 patients (65.2%) showed dysautonomic symptoms, 42 (91.3%) had parkinsonism, 10 (21.7%) had cerebellar symptomatology, and 8 subjects (17.4%) had corticospinal involvement. Although all were treated with levodopa, 43 of them (93.5%) showed no response to this treatment.

The [^123^I]Ioflupane SPECT test was performed on all subjects, with a result compatible with MSA in 93.5% of them and apparently normal in the remaining 6.5%. In the quantitative assessment of SPECT using the three S/O indices, the values obtained were: right S/O index $\overset{-}{x}$ = 1.34, SD = 0.12, (range: 1.14–1.63); left S/O index $\overset{-}{x}$ = 1.32, SD = 0.13, (range: 1.12–1.61); global S/O index $\overset{-}{x}$ = 1.33, SD = 0.13, (range: 1.13–1.60).

Scintigraphy with [^123^I]MIBG was also performed in all patients, 73.1% being compatible with MSA (normal uptake 58.7%; decreased uptake in 17.4%) and incompatible in the remaining 23.9% (absent uptake). The quantitative assessment of this test was performed using the delayed heart/mediastinum ratio, obtaining values of $\overset{-}{x}$ = 1.58, SD = 0.32, (range: 1.12–2.27).

The diagnostic effectiveness of both neuroimaging techniques was analyzed in the 46 patients with MSA (Table 1).

In the [^123^I]Ioflupane SPECT, the degree of agreement between the qualitative assessment (visual, performed by the nuclear physician) and the quantitative assessment (numerical, using the three S/O indices) was calculated (Table 2). Differences were found between both assessments when using the global S/O index to consider a patient as healthy or sick.

Similarly, in the [^123^I]MIBG scintigraphy, the relationship between both the visual assessment and numerical assessment was studied. After this, the results obtained by both techniques (SPECT and scintigraphy) were compared. Two groups were established according to the visual classification of the scintigraphy: “not suggestive of MSA” (subjects with no cardiac uptake) and “suggestive of MSA” (patients with normal or decreased cardiac uptake). We found differences between the qualitative and quantitative assessments of [^123^I]MIBG: “not suggestive of MSA” $\overset{-}{x}$ = 5500, SD = 4.035 vs. “suggestive of MSA” $\overset{-}{x}$ = 21.588, SD = 8.302; *p* < 0.001. However, we found no association between qualitative assessment of [^123^I]MIBG scintigraphy and quantitative [^123^I]Ioflupane SPECT by global S/O index: “not suggestive of MSA” $\overset{-}{x}$ = 0.921, SD = 0.601 vs. “suggestive of MSA” $\overset{-}{x}$ = 0.640, SD = 0.694; *p* > 0.05.

In addition, in order to determine the diagnostic performance of the [^123^I]MIBG technique, a logistic regression was performed between visual classification, numerical classification, and introducing as an additional variable the clinical diagnostic classification according to Gilman’s criteria (MSA-probable and MSA-possible). It was obtained that, for each unit increase in delayed HMR, the risk of suspected MSA increased by 1.58 units (OR = 1.58; CI (95%) [1.24–2.50]; *p* < 0.007).

Furthermore, the possible superiority of the visual assessment with each of the techniques (SPECT and scintigraphy) was analyzed. Two groups were formed: “suspected MSA” (when the scan result showed preserved or slightly diminished myocardial innervation), and “no suspected MSA” (when no/absent myocardial uptake was obtained on SPECT) (Table 3). Fisher’s test took a value of 1, indicating that neither technique showed superiority as no statistically significant association was observed between the two variables. Similarly, when analyzing the possible relationship between the qualitative assessment of the scintigraphy and the diagnostic classification according to Gilman’s criteria (Table 3), no significant differences were obtained (*p* < 0.47).

We wanted to know whether there was a possible correlation between the quantitative assessments of both studies ([^123^I]Ioflupane SPECT and [^123^I]MIBG scintigraphy). Spearman’s correlation coefficient was applied, obtaining a non-significant coefficient of r = 0.06 (*p* > 0.05), which indicated a very low correlation. 

**Figure 3:**
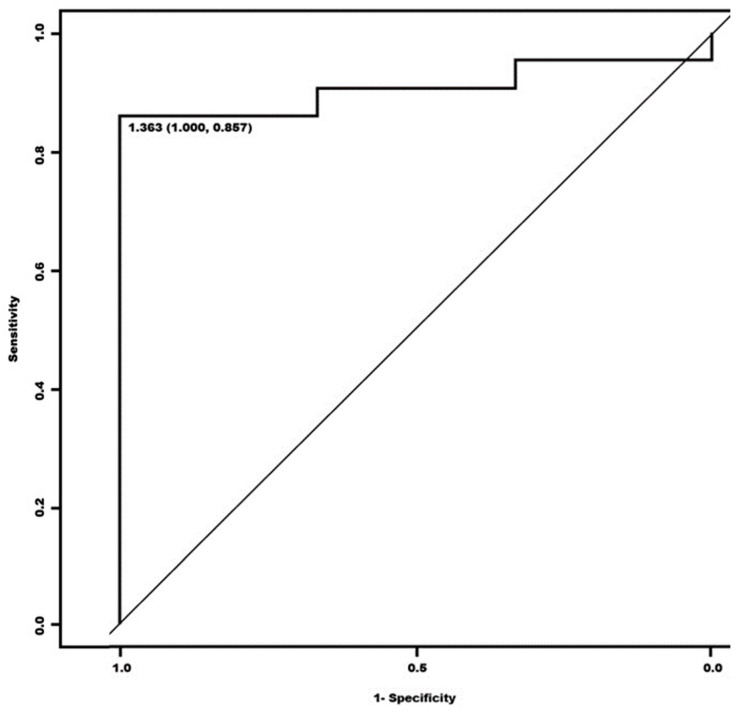
ROC curve of quantitative and visual assessment of the [^123^I]MIBG scintigraphy test for two categories (“suspected MSA”, “not suspected MSA”).

We calculated the optimal cut-off point within the quantitative assessment of the study with [^123^I]MIBG scintigraphy (delayed HMR). We obtained the ROC curve, whose graph showed an optimal cut-off of 1.43 with an S = 85.3% and E = 100% (Figure 3). The area under the curve (AUC) was 0.9647 (95% CI (0.918–1)).

**Figure 4:**
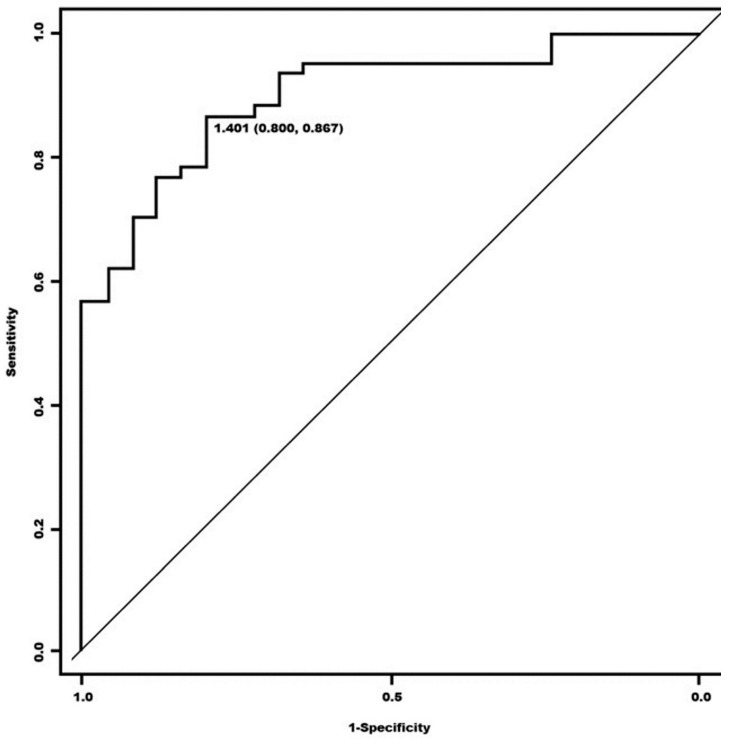
ROC curve of quantitative and visual assessment of the [^123^I]Ioflupane SPECT test for two categories (“pathological” versus “not pathological”).

Similar to a previous study by our group on the diagnostic effectiveness of [^123^I]Ioflupane SPECT in 139 patients with MSA, we obtained an ROC curve for the calculation of the cut-off of the global S/O index as a function of the SPECT visual assessment, according to two categories (“pathological” and “non-pathological”) (Figure 4). The optimal cut-off point was set at 1.363 (S = 85.7%, E = 100%) with an AUC of 0.9048 (95% CI (0.8099–0.9996)). This value is very close to that obtained in a study in which the cut-off was 1.401 (S = 86.7% and E = 80%). The AUC was 0.8951 (95% CI (0.8267–0.9635)).

## 4. Discussion

The present study analyzes the diagnostic performance of both neuroimaging tests in MSA, both independently and comparatively between them.

When analyzing the possible superiority of one technique over the other, we found no significant differences, so both techniques are equally useful in the diagnosis of MSA. In fact, no correlation could be established between the quantitative results of both tests. This may be since each of them evaluates different aspects affected in this pathology (dopaminergic nigrostriatal pathway and myocardial sympathetic innervation, respectively). However, it can also be deduced from this that, since they are not related, striatal and myocardial deterioration do not evolve in parallel, so we believe that both tests are necessary for an accurate diagnosis.

The [^123^I]Ioflupane SPECT was positive in 93.5% of subjects (S = 95.24%; PPV = 93.02%). Given this sensitivity value, we believe it wiser to perform this neuroimaging test first. The [^123^I]MIBG scintigraphy was compatible with MSA in 73.1% (S = 78.57%, PPV = 94.29%), which suggests that, together with the superior specificity (E = 50%) with respect to SPECT (E = 25%), performing this technique second would allow us to confirm selected patients and at the same time rule out possible false positives. Current guidelines recommend both techniques for the correct study of atypical parkinsonism [44]. In view of the above, we believe that the [^123^I]Ioflupane SPECT technique could be reconsidered in the diagnostic criteria for MSA, as it was in the previous review [10]. On the other hand, we also state the usefulness of [^123^I]MIBG scintigraphy as a support tool (and for the differential diagnosis of Parkinson’s disease) and the need for it to remain in the current criteria after the last revision [35]. In fact, once again when analyzing our variables with the previous diagnostic classification (when our study was performed), under the categories of MSA-probable and MSA-possible, we found no differences either in the final diagnosis or in the outcome of the neuroimaging tests. We think that with the creation/adaptation of the new categories after the last revision (Established and Clinically Probable MSA [35]), future studies will be able to check whether they are more accurate.

In this regard, the nuclear physician adequately classified a healthy subject from a diseased one in both neuroimaging techniques. Furthermore, in this study, we again confirm the relationship between the qualitative assessment of the nuclear physician and the quantitative assessment by means of specific uptake indexes as diagnostic support, in both tests. These findings are similar to previous research by our group [45].

As a first, we have shown that there is a direct relationship between the delayed HMR and the risk of MSA (for each unit increase in delayed HMR, the risk of suspected MSA increases by 1.58) (OR = 1.58, *p* < 0.05). In addition, we propose that the cut-off of this index could be set at 1.43 (S = 85.3, E = 100%), a value close to previous studies [46,47,48]. In addition, other neurodegenerative diseases such as PD show lower values with respect to this cut-off point, allowing us to differentiate between them [49,50]. 

As for the cut-off point in the [^123^I]Ioflupane SPECT, the overall S/O index was again shown to be the most appropriate for discerning, together with the qualitative assessment of the nuclear physician, between a healthy patient and a patient with MSA. It is worth noting that the cut-off obtained of 1.363 (S = 85.7%, E = 100%), from a significantly smaller number of cases than in previous studies, barely differs by 0.038 with respect to our previous study: 1.401 (S = 86.7% and E = 80%) [45].

Based on the results obtained, it could be thought that [^123^I]Ioflupane SPECT has a low specificity score and that the accuracy of [^123^I]MIBG scintigraphy is also low. One explanation for this finding may be that the patient sample obtained has been selected with strict inclusion and exclusion criteria, so that almost all patients have clinically confirmed MSA at follow-up (rather than pathology confirmation when the patients died). We have taken as a gold standard the clinical diagnosis at the end of the study period, where many of these patients had already died, so this is the definitive diagnosis. Therefore, when analyzing the results retrospectively, the diagnostic tests as a whole do not stand out for their specificity, but for their ability to detect (sensitivity) patients with a specific diagnosis (positive predictive value).

Therefore, it was assessed whether one test might be superior in diagnosing MSA compared to the other one, but no statistically significant differences were obtained. This was expected, as both studies focus on different pathophysiological pathways affected in MSA. Consequently, neither test can replace the other; rather, they complement each other. In order to identify a movement disorder, neurologists usually first request [^123^I]Ioflupane SPECT and can thus confirm that a degenerative process is present (instead of other forms of parkinsonisms, such as those with pharmacological causes). Secondly, they request [^123^I]MIBG scintigraphy to make a differential diagnosis of PD or Lewy body dementia versus other atypical parkinsonisms (MSA, progressive supranuclear palsy, or corticobasal degeneration). These findings support the results obtained in our previous research, in which we advocated for the need to include both imaging studies in the diagnostic algorithm of MSA [36,45].

The novelty of this study lies in the direct comparison between both imaging tests in the specific case of multiple system atrophy, where the specific literature on the subject is lacking. In fact, in the field of nuclear medicine, data such as those provided by this study are necessary to establish a cut-off point in the quantitative analysis of [^123^I]MIBG scintigraphy. For this reason, we point out what information is provided by each of the two neuroimaging tests in MSA.

Regarding the weaknesses and strengths of this research, we could denote the limitations of a cross-sectional study. If we had followed up with the recruited patients, then we could have verified whether the diagnostic effectiveness of both neuroimaging tests (in terms of sensitivity and specificity) was maintained over time, or whether, on the contrary, in subsequent analyses, errors in the diagnostic classifications were detected and the previous results would have to be reconsidered. For example, we could have known how many cases of MSA-probable and MSA-possible were confirmed in the long term. By following up with patients, we could have serially observed changes in both forms of multiple system atrophy (MSA-C and MSA-P), both with [^123^I]Ioflupane SPECT and [^123^I]MIBG scintigraphy. However, although the recruitment period was long, it was not feasible to perform an evolutionary study because MSA has a poor prognosis, and because of this, many of the patients (or their relatives) wished to drop out of the study when they were aware of the denouement of the disease; other patients died within a short period of time. Moreover, based on our hospital protocol, when the patient already has an established diagnosis of MSA, it is not appropriate to perform both neuroimaging techniques. Consequently, we were only able to follow up with a subsample of these patients with one of the two techniques, namely [^123^I]Ioflupane SPECT [36].

Another possible limitation is that a stratified analysis by groups was not performed. For example, dividing by age and gender, to try to determine differences in the severity of MSA in the different groups. Another interesting possible subdivision would be based on the presence of dysautonomic symptoms, parkinsonism, cerebellar symptoms, or corticospinal involvement. However, when subdividing by groups, the size of each group was very small, and the statistical results were not reliable. In this regard, we must keep in mind that MSA has a very low prevalence, so achieving large sample sizes is very difficult.

It could be noted as a limitation of the present study that it is a single-center study. If a multicenter study had been carried out, then the results would have had a higher external validity. This would also have made a larger sample size possible. However, if a multi-hospital study had been performed, then we would have to take into account the possible inter-observer variability. In our investigation, all neuroimaging tests were evaluated by the same group of nuclear medicine physicians (all diagnostic reports were made by consensus among the three participating physicians). Consequently, there was no variability among the imaging assessments, which confers reliability to the results in this aspect.

## 5. Conclusions

Both imaging techniques ([^123^I]Ioflupane SPECT and [^123^I]MIBG scintigraphy) have been shown separately to be useful in the diagnosis of MSA, with optimal diagnostic performance. Comparatively, we observed no net superiority of either. This, together with the fact that striatal and myocardial deterioration do not seem to evolve in parallel, supports the need to consider both tests in the diagnosis of MSA. The qualitative assessment of the nuclear physician remains crucial in both techniques, together with the support of quantitative analysis using established indices and our proposed cut-off. Delayed HMR shows a direct relationship with the risk of MSA.

## Figures and Tables

**Table 1:** Diagnostic effectiveness of both techniques ([^123^I]Ioflupane SPECT and [^123^I]MIBG scintigraphy) in MSA.

	[^123^I]Ioflupane SPECT	[^123^I]MIBG Scintigraphy
Sensitivity	95.24% (87.61–100)	78.57% (64.97–92.17)
Specificity	25% (0.00–79.93)	50% (0.00–100)
PPV	93.02% (84.25–100)	94.29% (85.17–100)
PNV	33.33% (0.00–100)	18.18% (0.00–45.52)
Accuracy	89.13%	76.09%

Percentage values (%) ± 95% confidence interval; PPV: positive predictive value; PNV: negative predictive value.

**Table 2:** Association between qualitative and quantitative SPECT assessments in two groups (healthy and sick).

S/O Indexes	Healthy	Sick	*p* Value
Global	$\overset{-}{x}$ = 1.429 (S = 0.056)	$\overset{-}{x}$ = 0.658 (S = 0.672)	*
Right	$\overset{-}{x}$ = 1.421 (S = 0.063)	$\overset{-}{x}$ = 1.329 (S = 0.127)	>0.05
Left	$\overset{-}{x}$ = 1.437 (S = 0.056)	$\overset{-}{x}$ = 1.304 (S = 0.133)	>0.05

$\overset{-}{x}$: average scores; SD: standard deviation; * *p* < 0.05.

**Table 3:** Association of qualitative assessment of [^123^I]Ioflupane SPECT or qualitative assessment of [^123^I]MIBG scintigraphy in both groups: “suspected MSA” and “non-suspected MSA”.

		Visual Scintigraphy *		
		Suspected MSA	Non-Suspected MSA	*p* Value
Visual SPECT **	Negative 3 (6.5%)	3 (8.6%)	0	*p* > 0.05
	Positive 43 (93.5%)	32 (91.4%)	11 (100%)	
Diagnostic classification	Possible 13 (28.3%)	11 (31.4%)	2 (18.2%)	*p* > 0.05
	Probable 33 (71.7%)	24 (68.6%)	9 (81.8%)	

(*) Visual evaluation of [^123^I]MIBG scintigraphy (qualitative classification); (**) visual evaluation of [^123^I]Ioflupane SPECT (qualitative classification).

## Data Availability

Data is contained within the article.

## Abbreviations

CI = Confidence Interval; E = Specificity; HMR = Heart/Mediastinum Ratio; MIBG = meta-iodobenzylguanidine; MSA = Multiple System Atrophy; MSA-P = Multiple System Atrophy Parkinsonian Type; MSA-C = Multiple System Atrophy Cerebellar Type; ROI = Region of Interest; S = Sensitivity; SD = Standard Deviation; S/O index = Striatum/Occipital index; SPECT = Single-Photon Emission Computed Tomography.
